# Why I Want Bunion Surgery—the Patient’s Preoperative and Postoperative Perspective

**DOI:** 10.1177/10711007251321475

**Published:** 2025-04-04

**Authors:** Michael de Buys, Nikiforos P. Saragas, Paulo N.F. Ferrao

**Affiliations:** 1 Orthopaedic Foot and Ankle Unit, Linksfield Hospital, Johannesburg, South Africa; 2 Division of Orthopaedic Surgery, School of Clinical Medicine, Faculty of Health Sciences, University of the Witswatersrand, Parktown, Johannesburg, South Africa

**Keywords:** hallux valgus, bunion, PROM

## Abstract

**Background:**

Hallux valgus is the most common pathology afflicting the hallux. Surgery is generally offered to symptomatic patients who fail conservative treatment. The aim of this study is to evaluate patient-reported reasons for undergoing hallux valgus corrective surgery in a preoperative and postoperative cohort.

**Methods:**

We performed a prospective and retrospective cross-sectional study. Our study included all patients aged >18 years who were planning to or have had hallux valgus surgery during the study period. An information sheet including 14 possible reasons for having hallux valgus surgery and a questionnaire to rank each reason (1-10) was sent to all patients. Patients were divided into a preoperative group and a postoperative group to eliminate bias. In our study we had 101 patients, 5 males and 96 females, at an average age of 50.6 years.

**Results:**

The preoperative cohort included 51 patients and the postoperative cohort 50 patients. The 3 most important reasons for having surgery, in both cohorts, were the ability to move pain free, eliminate pain over bunion, and to be able to walk long distance and over uneven terrain without pain. The 3 least important factors were to reduce the need for an orthotic, narrower foot, and to wear high heels. Indications were divided into one of 4 categories. Pain was the highest-rated category in both pre and postoperative groups, followed by function. In the preoperative group, appearance was the next most important group and shoe wear the least. In comparison, in the postoperative group, shoe wear was more important than appearance.

**Conclusion:**

Pain and function are the most important reasons patients have for surgery in both the preoperative and postoperative patient cohorts. Patients were more likely to list cosmesis as a reason to undergo surgery in the preoperative than the postoperative group.

**Level of Evidence:** Level III, retrospective cohort study.

## Introduction

Hallux valgus, a multiplanar deformity, is the most common pathology afflicting the hallux.^3^ There is a strong female predilection with a reported female-to-male ratio of 14:1.^10^ The overall accumulative prevalence of hallux valgus is estimated at 7.8% in juveniles, 23% in adults aged 18-65 years, and 35.7% in persons aged >65 years.^10,12^

Conservative measures, aimed at reducing pain and symptoms, are viable options as initial treatment options. However, these are ineffective in correcting the deformity, and surgical correction is ultimately offered to those who fail to respond to conservative treatment. More than 100 surgical procedures have been described for hallux valgus correction and algorithms are typically used to select suitable surgical procedures utilizing objective radiographic measurements.^7^ The implementation of different strategies are usually based on a surgeon’s training and preference, leading to great variability in assessing outcome measures.^9^

Clinical studies have predominantly reported on outcomes by surgeons themselves, whether it be clinical or radiologic. In order to stay abreast with value-based orthopaedics, clinical specialists strive to demonstrate that treatment delivers an improvement of clinical importance using measures that are derived from the patient’s perspective. This growing interest in patient-reported outcomes in orthopaedic surgery includes the patients’ report on pain, function, activity and satisfaction.^5^ Substantial investigation exploring the relationship between patient expectation and satisfaction has been performed.^4,6^ To improve patient satisfaction and avoid disappointment following surgery, the patient expectations should be thoroughly understood.^1,5^ Identifying patients with high and possibly unachievable expectations and addressing these expectations in the preoperative setting may prevent poor patient-reported outcomes.

Hallux valgus surgery for purely cosmetic reasons is typically discouraged as surgery has potential risks that may ultimately adversely affect an asymptomatic foot. Studies have reported that cosmesis, although among the most volunteered reasons for undergoing hallux valgus surgery, are actually lower on the list of prioritized reasons.^1,8,12,13^

Although pain relief tends to be the predominant outcome in hallux valgus surgery, there are other expectations that may be more important for certain patients.^1,3,9,12^ Up to 33% of patients remain dissatisfied with surgical outcomes despite improvement in radiologic parameters and decreased pain suggesting a discrepancy between patient appraisal of results and the clinical outcome.^13^ MacMahon et al^8^ found that more than two-thirds of patients had higher overall expectations than did their surgeons. Patients were evidently more optimistic about the surgical outcome and many patients may have been poorly informed of what to expect, leading to worse satisfaction when these high expectations were not met.^8^

The aim of this study is to evaluate patient-reported preoperative and postoperative reasons for undergoing hallux valgus corrective surgery. We hypothesized that patients report functional and pain-related reasons preoperatively, while consciously or subconsciously cosmesis played an important role in the decision making. Postoperatively the patient may be more comfortable in listing cosmesis as a main reason for having surgery; if true, this would make meeting patient expectations difficult, likely resulting in poorer patient-reported outcomes.

## Patients and Methods

We performed a prospective and retrospective cross-sectional study from June 2020 to May 2022. Our study included all patients aged >18 years who were booked for or have had hallux valgus surgery during this study period. All surgeries were performed by one of 2 fellowship-trained foot and ankle surgeons. Exclusion criteria included recurrent or revision hallux valgus surgery and patients with rheumatoid arthritis, or neuromuscular pathology. Importantly, our unit does not offer elective foot and ankle hallux valgus correction to active smokers or diabetic patients with an HbA_1c_ of >7.5%. The preoperative cohort included 51 patients and the postoperative cohort 50 patients. Of the 101 patients included in the study, we had 5 male patients and 96 females; 2 males and 49 females in preoperative cohort and 3 males and 47 females in postoperative cohort). The average age of our study population was 50.6 years (average age in preoperative and postoperative cohort was 52.04 years and 49.06 years, respectively). Hallux valgus angles were calculated in each cohort preoperatively and averaged with results as follows:

- Preoperative cohort:
  - Hallux valgus angle = 29.5 degrees (22.8-33.6 degrees)
  - Intermetatarsal angle = 15.4 = degrees (13.3-20.2 degrees)
  - Distal metatarsal articular angle = 8.2 degrees (4.3-11.5 degrees)

- Postoperative cohort
  - Hallux valgus angle = 32.1 degrees (23. 9-36.1 degrees)
  - Intermetatarsal angle = 13.8 degrees (9.6-17.1 degrees)
  - Distal metatarsal angle = 9.1 degrees (5.1-16.2 degrees)

This study was approved by the local Human Ethics Committee (ethics approval no: M191167).

An information sheet and questionnaire was sent to all patients and completed either by email or an electronic survey platform that uses anonymous electronic data collection. We divided our study group into two: a preoperative and a postoperative group. The preoperative group included patients who completed the information sheet before surgery and were therefore questioned prospectively. The postoperative group completed the information sheet a minimum of 3 months after surgery and were therefore questioned retrospectively. A patient could only be in one cohort, and this was done to eliminate bias; we postulated that if a patient is in both cohorts he or she may be hesitant to change his or her postoperative indications on account of one’s awareness of being part of a study. We included the first 50 patients during this study period for both cohorts, that is, surgery performed during the period or postoperative follow-up during the period. There was no overlap between the 2 groups.

The information sheet included 14 possible reasons for having hallux valgus surgery. The patient was asked to rate each reason according to its contribution to requesting the corrective surgery. A score of 10 indicates the most important reason for having surgery, whereas 0 indicates the reason that did not play a role in the decision making. To avoid bias, terms like cosmesis and aesthetics were avoided. The reasons for surgery were worded to cover 4 main groups: pain, dysfunction, cosmesis, and aids/shoewear.

## Results

**Figure 1.**
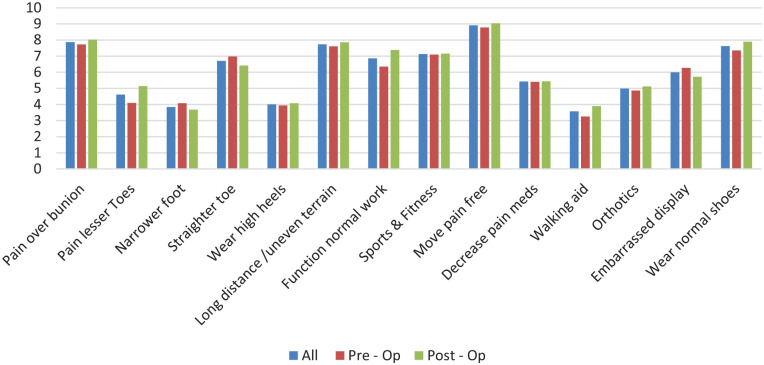
All reasons for surgery on questionnaire plotted against the average rating (0-10) for preoperative patients, postoperative patients, and combined (all).

Each reason for surgery on the given questionnaire and the average rating of importance is graphically compared in Figure 1. Average values for all and each cohort (preoperative and postoperative) are depicted.

When assessing the 2 cohorts together, the 3 most important reasons for surgery (in order of importance) were the ability to mobilize pain free, eliminate pain over the bunion, and be able to walk long distance/over uneven terrain without pain. The 3 least important factors were to reduce the need for an orthotic/walking aid, for a narrower foot, and wearing high heels.

When comparing the reasons for surgery between the pre- and postoperative cohorts minor differences do exist, but the major reasons remain the same. In both groups, the 4 most important reasons for patients wanting surgery are the same, including the order of importance (the ability to move pain free, to eliminate the pain over the bunion, to be able to walk long distance and over uneven terrain, and use normal footwear). Furthermore, the 5 least rated reasons (reduce the need for a walking aid, narrower foot, to wear high heels, painful lesser toes and to remove orthotics) were similar in both groups; the order did change, with the least important reason in the preoperative cohort being to reduce the need of an orthotic/walking aid vs the desire for a narrower foot in the postoperative cohort.

**Figure 2.**
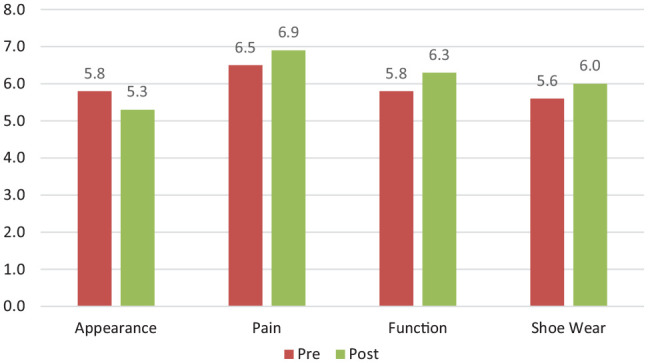
Categorization of reasons for requesting surgery (*x* axis) and comparison of the average level of importance (scale of 1-10) (*y* axis) of each between the pre- and postoperative cohorts.

Indications were then divided into one of 4 categories: appearance (narrower foot, straighter toe, embarrassed when feet on display), pain (pain over bunion, pain over lesser toes, move pain free, decrease pain medication), function (walk long distance or over uneven terrain, function normally at work, participate in sports and fitness, reduce need for walking aid, reduce need for orthotic) and shoe wear (wear high heels, wear normal shoes). Pre- and postoperative comparisons were done between each category and represented in Figure 2. Pain was the highest-rated category in both pre- and postoperative groups, followed by function. In the preoperative group, appearance was the next most important group and shoe wear the least as compared to the postoperative group, where shoe wear was more important than appearance.

## Discussion

We endeavored to evaluate patient-reported reasons for undergoing hallux valgus corrective surgery. Tai et al^13^ assessed preoperative patient expectations for wanting hallux valgus surgery and reported that improved walking, pain relief, and shoe wear to be the most important expectations preoperatively. By only questioning the patients preoperatively, their study may have an inherent bias in that patients may be reluctant to choose aesthetic reasons for having corrective surgery. Our study has demonstrated that pain and function are the most important reasons for surgery in both the preoperative and postoperative patient cohorts. Pain and function are inextricably linked, and this is evident in our results. It is interesting to note that appearance was seen as a more important factor than shoe wear in our preoperative group, but this was reversed in the postoperative group where shoe wear was more important. It is possible that patients preoperatively may not fully understand the risk of surgery, the pain associated with foot and ankle procedures, the required rehabilitation, and the postoperative impact on activities of daily living. Postoperative patients may therefore develop improved insight into the surgical experience and therefore what they would constitute an acceptable reason for surgery (eg, if they require contralateral surgery).

In a large heterogeneous cohort of foot and ankle pathologies, Al-Mohrej et al^1^ found that patients who expected improvement related to pain, walking ability or shoe wear were generally satisfied. However, those who had undergone surgery for cosmetic reasons were less satisfied.^1^ Cody et al^2^ confirmed these findings and determined that the preoperative diagnosis impacts expectation and satisfaction significantly. They reported that women have 1.5 times higher expectation of complete improvement than men and that expectation of improved appearance was most prevalent among hallux valgus patients.^2^ Schneider and Knahr^12^ assessed expectations in patients with hallux valgus and their surgeons. They found that the most important expectation for patients were pain relief, walking distance, and the ability to wear conventional shoes.^12^ From a surgeon’s perspective, they found metatarsophalangeal range of motion to be the most important variable.^12^ Pouliart et al^11^ analyzed a cohort of patients undergoing hallux valgus surgery and reported that 90% of their cohort were satisfied with their outcomes despite 23% of feet still having a radiographic hallux valgus angle greater than 30 degrees, suggesting that there is more to satisfaction than radiographic correction.

We are the first study that has compared preoperative and postoperative patient-reported indications for requesting hallux valgus surgery. Our study was structured to have separate preoperative and postoperative cohorts as we suspected patients might be more forthcoming with the reason for requesting hallux valgus surgery after the fact; especially regarding the importance of cosmesis. Our study did have limitations. Certain parameters analyzed in our study would be specifically important only in certain cohorts. Reducing the need for orthotics was noted to be one of the least important reasons to undergo hallux valgus surgery in our patient sample. However, it is not discussed how many of these patients actually required or used orthotics preoperatively. It would be relevant to identify the importance of this in a subset including only patients who use orthotics. This concept extends into other parameters, for instance, the desire to wear high heels. Our study consisted mostly of women (a male-female ratio of 1:20), which is in keeping with reported incidence of hallux valgus deformity. A future study of an only-male cohort could give us further insight as to the reasons a male patient would request surgical correction, perhaps differing from female patients. In addition, the influence of socioeconomic status as a factor would be important to look at.

Our hypothesis that postoperatively patients would rate cosmesis as an important indication for having hallux valgus correction has been disproven. Importantly, this confirmed the conclusion found in preoperative studies that although cosmesis is a reason patients undergo hallux valgus surgery, it has a lower priority compared with pain, function, and shoe wear.^1,8,12,13^ We as surgeons often focus on radiographic and biometric parameters rather than patient-reported expectations. As Pouliart et al^11^ reported, we need to take patient expectations into consideration when defining the success of a procedure. It is critical in the preoperative period to explore the expectations, concerns, needs and desires from the patient and be clear and honest about what can be achieved with surgery. We believe a clear understanding of expectations between the patient and the surgeon is crucial for a positive outcome.

## Conclusion

We demonstrate that pain and function are the most important reasons for patients requesting hallux valgus surgical correction. Although our suspicion was that certain patients request surgery for cosmetic reasons, our data does not support this. We believe our study forms an important link in highlighting the importance of patient expectations in the management of hallux valgus deformities.

## Supplemental Material

sj-docx-2-fai-10.1177_10711007251321475 – Supplemental material for Why I Want Bunion Surgery—the Patient’s Preoperative and Postoperative Perspective Supplemental material, sj-docx-2-fai-10.1177_10711007251321475 for Why I Want Bunion Surgery—the Patient’s Preoperative and Postoperative Perspective by Michael de Buys, Nikiforos P. Saragas and Paulo N.F. Ferrao in Foot & Ankle International

sj-pdf-1-fai-10.1177_10711007251321475 – Supplemental material for Why I Want Bunion Surgery—the Patient’s Preoperative and Postoperative Perspective Supplemental material, sj-pdf-1-fai-10.1177_10711007251321475 for Why I Want Bunion Surgery—the Patient’s Preoperative and Postoperative Perspective by Michael de Buys, Nikiforos P. Saragas and Paulo N.F. Ferrao in Foot & Ankle International
