# Supplementary material for: Why I Want Bunion Surgery—the Patient’s Preoperative and Postoperative Perspective
Source: Foot Ankle Int. 2025 Apr 4;46(4):410–4. doi: 10.1177/10711007251321475 (PMC12003933; doi:10.1177/10711007251321475)
Supplement: sj-docx-2-fai-10.1177_10711007251321475 – Supplemental material for Why I Want Bunion Surgery—the Patient’s Preoperative and Postoperative Perspective [file sj-docx-2-fai-10.1177_10711007251321475.docx]

Questionnaire:

With regards to why you felt you wanted to undergo bunion surgery, please mark each option individually on a scale from 1 – 10 (l – not important; 10 – very important). Please note that one or more option may be allocated the same level of importance i.e. the same number.

| I have pain over the bunion  I have pain over my lesser toes  I want a narrower foot  I want a straighter toe  I want to be able to wear high heels again  I want to improve my long distance and uneven terrain walking  I want to function normally at work  I want to partake in sports and fitness activities  I want to move around pain free  Reduce the need for pain medication  Reduce the need for walking aid (e.g. Crutch)  Reduce the need for orthotics  I am embarrassed to display feet in public (e.g. Beach/pool)  I want to wear normal (non-orthopaedic) shoes |  |
| --- | --- |
